# The Essential Oil and Hydrolats from *Myristica fragrans* Seeds with Magnesium Aluminometasilicate as Excipient: Antioxidant, Antibacterial, and Anti-inflammatory Activity

**DOI:** 10.3390/foods9010037

**Published:** 2020-01-02

**Authors:** Inga Matulyte, Aiste Jekabsone, Lina Jankauskaite, Paulina Zavistanaviciute, Vytaute Sakiene, Elena Bartkiene, Modestas Ruzauskas, Dalia M. Kopustinskiene, Antonello Santini, Jurga Bernatoniene

**Affiliations:** 1 Department of Drug Technology and Social Pharmacy, Lithuanian University of Health Sciences, LT-50161 Kaunas, Lithuania; inga.matulyte@lsmuni.lt (I.M.); jurga.bernatoniene@lsmuni.lt (J.B.); 2 Institute of Pharmaceutical Technologies, Medical Academy, Lithuanian University of Health Sciences, LT-50161 Kaunas, Lithuania; aiste.jekabsone@lsmuni.lt (A.J.); lina.jankauskaite@lsmuni.lt (L.J.); daliamarija.kopustinskiene@lsmuni.lt (D.M.K.); 3 Department of Pediatrics, Lithuanian University of Health Sciences Hospital Kauno Klinikos, LT-50161 Kaunas, Lithuania; 4 Department of Food Safety and Quality, Lithuanian University of Health Sciences, LT-47181 Kaunas, Lithuania; paulina.zavistanaviciute@lsmuni.lt (P.Z.); vytaute.sakiene@lsmuni.lt (V.S.); elena.bartkiene@lsmuni.lt (E.B.); 5 Institute of Microbiology and Virology, Lithuanian University of Health Sciences, LT-47181 Kaunas, Lithuania; modestas.ruzauskas@lsmuni.lt; 6 Department of Pharmacy, University of Napoli Federico II, Via D. Montesano 49, 80131 Napoli, Italy

**Keywords:** nutmeg, essential oil, antioxidant activity, antibacterial activity, poly I:C-induced inflammation, fibroblasts, magnesium aluminometasilicate

## Abstract

Nutmeg (*Myristica fragrans*) essential oil has antimicrobial, antiseptic, antiparasitic, anti-inflammatory, and antioxidant properties. We have recently demonstrated that hydrodistillation of nutmeg essential oil by applying magnesium aluminometasilicate as an excipient significantly increases both the content and amount of bioactive substances in the oil and hydrolats. In this study, we aimed to compare the antioxidant, antimicrobial, and anti-inflammatory activity of hydrolats and essential oil obtained by hydrodistillation in the presence and absence of magnesium aluminometasilicate as an excipient. The 2,2-diphenyl-1-picrylhydrazyl (DPPH) radical scavenging method revealed that magnesium aluminometasilicate did not significantly improved antioxidant activity of both essential oil and hydrolat. Antibacterial efficiency was evaluated by monitoring growth of 15 bacterial strains treated by a range of dilutions of the essential oil and the hydrolats. Essential oil with an excipient completely inhibited the growth of *E. faecalis*, *S. mutans* (referent), and *P. multocida*, whereas the pure oil was only efficient against the latter strain. Finally, the anti-inflammatory properties of the substances were assessed in a fibroblast cell culture treated with viral dsRNR mimetic Poly I:C. The essential oil with an excipient protected cells against Poly I:C-induced necrosis more efficiently compared to pure essential oil. Also, both the oil and the hydrolats with aluminometasilicate were more efficient in preventing IL-6 release in the presence of Poly I:C. Our results show that the use of magnesium aluminometasilicate as an excipient might change and in some cases improve the biological activities of nutmeg essential oil and hydrolats.

## 1. Introduction

Since ancient times, *Myristica fragrans* (nutmeg) seeds have been used as a food spice, flavoring agent, a natural remedy for headaches and fever [1]. Nutmeg seeds have essential and fatty oils, resins, wax, and other components [2]. Nutmeg’s essential oil has antimicrobial, antiseptic, antiparasitic, anti-inflammatory, and antioxidant properties [1,3]. The concentration of essential oil in nutmeg seeds is about 5–15% [4], and its major components are terpene hydrocarbons (sabinene, pinene, camphene, *p*-cymene, phellandrene, terpinene, limonene, and myrcene altogether make up 60% to 80% of the oil), oxygenated terpenes (linalool, geraniol, and terpineol, which make up approximately 5% to 15%) and aromatic ethers (myristicin, elemicin, safrole, eugenol, and eugenol derivatives, together constituting 15 to 20%) [5,6,7,8]. The toxicity of nutmeg seeds at high doses has been reported, mainly due to myristicin oil and elemicin, causing tachycardia, nausea, vomiting, agitation, and hallucinations. However, these effects are related to the abuse of the spice and are not observed at usual low concentrations [9]. There are many studies on the beneficial effects of nutmeg seed and various nutmeg seed extracts. One of the most prominent biological activities of the nutmeg preparations is antibacterial. Nutmeg seed lignans exert antimicrobial activity on *Bacillus subtilis*, *Staphylococcus aureus*, and *Shigella dysenteriae* [10]. Ethanol and acetone extracts of nutmeg crust have strong antibacterial activity against gram-positive bacteria *Staphylococcus aureus* [5]. Ethyl acetate extracts of flesh of the nutmeg fruit have inhibitory potential against both gram-positive and gram-negative bacteria with a minimum inhibitory concentration (MIC) ranging from 0.625 to 1.25 mg/mL [11]. Used for the preservation of sweets, nutmeg methanol extracts inhibit growth of *Staphylococcus aureus*, *Aspergillus niger*, *Saccharomyces cerevisiae*, and *Escherichia coli* at MIC between 250 and 300 mg/mL [12]. However, there are only a few studies on the biological activity of nutmeg essential oil. Takikawa et al. showed a higher antibacterial effect of essential nutmeg oil on pathogenic compared to non-pathogenic strains of *Escherichia coli* [13]. Furthermore, nutmeg essential oil decreased the growth and survival of *Yersinia enterocolitica* and *Listeria monocytogenes* in broth culture [14].

Nutmeg oil preparations are also known for their antioxidant capacity. Using the 2,2-diphenyl-1-picrylhydrazyl (DPPH) free radical scavenging assay, Piaru et al. reported a significant antioxidant activity of nutmeg oil [15]. The antioxidant properties are often related to the alleviation of inflammation. Nutmeg oil diminished chronic inflammation and pain through the inhibition of COX-2 expression and substance P release in vivo [16]. In another study, nutmeg oil suppressed reactive oxygen species (ROS) production in human neutrophils stimulated by PMA (phorbol 12-myristate 13-acetate) [17] and mildly inhibited phagocytosis in human neutrophils [18]. However, there is no published research on the effect of nutmeg seed essential oil on virus-triggered inflammatory response.

Hydrodistillation is a popular method used for the preparation of essential oils. However, hydrodistillation with excipients is not widely used—we have found just three studies applying this method so far [19,20,21]. Therefore, we have applied magnesium aluminometasilicate in hydrodistillation as the new excipient and have tested its effects on the nutmeg essential oil yield and its composition [22]. Aluminometasilicate is widely used as a disintegrator in the manufacturing of tablets. Furthermore, this compound is non-toxic and inexpensive, as the price is ~300 eur for 25 kg. Magnesium aluminometasilicate has significantly increased both the yield and composition of some chemical compounds (sabinene, α-pinene, and limonene). The use of the excipient also increased the essential oil yield by about 61% (hydrodistillation with water—the yield is 0.79 ± 0.04 g, using 1% excipient—1.29 ± 0.05 g; the nutmeg quantity was 15 g, the water content was 300 mL) [22].

The increased amount of active substances suggests that oil preparations with aluminometasilicate might have stronger biological activities. Therefore, in this study we compared the antioxidant, antimicrobial, and anti-inflammatory properties of *Myristica fragrans* seed essential oil preparations with and without aluminometasilicate.

## 2. Materials and Methods

### 2.1. Plant Material

The dried seeds of nutmeg (*Myristica fragrans*) were from Grenada. Seeds were identified by Jurga Bernatoniene, Medical Academy, Lithuania University of Health Sciences, Kaunas, Lithuania. A voucher specimen (I 18922) was placed for storage at the Herbarium of the Department of Drug Technology and Social Pharmacy. The seeds had a characteristic odor, a strong, bitter, and spicy flavour, and they were a brown-beige color. The seeds were ground into a powder (using laboratory mill), with particles smaller than 0.5 mm. All powder samples were kept in a dark and airtight container at 20 ± 2 °C.

### 2.2. Essential Oil and Hydrolat

The essential oil from nutmeg seeds was prepared by using hydrodistillation. The modified Clevenger type apparatus was used. Two samples of essential oil were prepared: one without excipient and the other with 1% of magnesium aluminometasilicate. Each sample was prepared with 15 g of nutmeg powder and 300 mL distilled water, and 1% magnesium aluminometasilicate was used as an excipient in one of the samples. Also, hydrolat of these two essential oils was used. It was collected from Clevenger apparatus. This material was collected first, followed by the essential oil. All samples were obtained and stored in airtight bottles in the refrigerator. The hydrodistillation took 4 h.

### 2.3. Antioxidant Activity by DPPH Radical Scavenging Assay

Antioxidant activity of nutmeg essential oil and hydrolat were evaluated using DPPH (Sigma Aldrich, St. Louis, MO, USA) [15]. First of all, 0.1 mM 96% DPPH solution in 96% ethanol was prepared. A total of 1 mL of DPPH solution was placed in a spectrophotometer cuvette and 100 µL of ethanolic essential oil solution at concentrations ranging from 0.2% to 20% was added. For an antioxidant activity evaluation of hydrolat, the absolute hydrolat was used. 1 mL DPPH solution and nutmeg hydrolat from 0.1 mL to 1 mL were mixed in a cuvette. All samples were incubated in the dark for 20 min and absorbance was taken at 515 nm. The antioxidant activity was performed on a UV Spectrophotometer UV-1800 (Shimadzu, Kyoto, Japan). The quantity of DPPH radical scavenging activity was calculated by using this formula:

(1) $$DPPH\;scavenging\;effect\;\%=\frac{A_{control}-A_{sample}}{A_{control}}\times 100,$$

where *A_control_* and *A_sample_* are the absorbance of the control sample (0.1 mM DPPH solution, solvent is 96% ethanol) and the experiment sample.

### 2.4. Antimicrobial Activity

The method used for antimicrobial activity was serial dilutions in liquid medium [23]. The broth liquid medium was dispensed into test tubes to give a final volume of 10 mL (with a sample of essential oil). The medium was sterilized. The physiological solution was dispensed into 5 mL individual tubes and used for preparation of suspension of the following bacteria: *Klebsiella pneumoniae*; *Salmonella enterica* 24 SPn06; *Pseudomonas aeruginosa* 17–331; *Acinetobacter baumanni* 17–380, *Proteus mirabilis*; 6MRSA M87fox; *Enterococcus faecalis* 86; *Enterococcus faecium* 103; *Bacillus cereus* 18 01; *Streptococcus mutans* (referent); *Enterobacter cloacae*; *Citrobacter freundii*; *Staphylococcus epidermidis*; *Staphylococcus haemolyticus*; *Pasteurella multocida strains.* All bacteria were isolated from clinical material. For each bacterial culture, three tubes of Mueller Hinton broth were used (9.94 mL, 9.97 mL, and 9.98 mL each). The tubes were inoculated with 10 μL of bacterial suspension with the essential oil at concentration 0.1%, 0.2%, and 0.5%. After 48 h of incubation, each tube was inoculated with 10 μL of suspension on soy-tryptone agar (Thermo Fisher, Hampshire, UK). MIC of essential oil was evaluated based on the presence of bacterial growth (bacterial colonies growing (+)/non growing (−).

### 2.5. Cell Culture and Treatments

Human fibroblasts (BJ-5ta, hTERT, LGC Standards Ltd. Middlesex, UK) were grown in 75 cm^2^ flasks in Dulbecco’s Modified Eagle Medium (DMEM) with Glutamax (Thermo Fisher Scientific, Waltham, MA, USA), 10% fetal bovine serum and 100 IU/mL Penicilin/Streptomycin according to standard supplier protocol. At 70–90% confluence, the cells were detached by 0.025% Trypsin/EDTA and plated in 96 well plates at a density of 2 × 10^5^ cells/well. A total of 24 h after plating, the cells were treated with 1 µg/mL Poly I:C to simulate viral dsRNR-induced inflammatory response. For cell culture treatments, the essential oils were dissolved in 96% ethanol at a concentration of 5% (*v*/*v*). For determination of cell viability and LD_50_, the solutions of essential oils and absolute hydrolats were used as a range of dilutions in cell culture medium starting from essential oil preparation to a medium *v*/*v* ratio of 1:1000 and finishing with 1:5. For the control, the same dilutions with solvent (ethanol) were performed. For anti-inflammatory activity evaluation, the solutions of essential oils at *v*/*v* dilutions of 1:100 or 1:200, or absolute hydrolats at *v*/*v* dilutions of 1:40, 1:100, and 1:200 were applied simultaneously with Poly I:C treatment.

### 2.6. Determination of Cell Viability and Determination on LD_50_

Cell viability was assessed by using double nuclear fluorescent staining with Hoechst 33342 (10 µg/mL) and propidium iodide (PI, 5 µg/mL) according to standard supplier protocol for 5 min at 37 °C. PI-positive nuclei indicating lost nuclear membrane integrity were considered to be necrotic. Cells were visualized under fluorescent microscope OLYMPUS IX71S1F-3, counted in fluorescent micrographs and expressed as percentage of total cell number per image. The data is presented as averages ± standard deviation. LD_50_ was calculated by SigmaPlot v.13 (Systat Software Inc., San Jose, CA, USA) using the equation selected by a dynamic curve fitting tool.

### 2.7. Assessment of Interleukin-6 Concentration

Medium collected after cell culture treatments was used to measure the concentration of pro-inflammatory cytokine interleukin-6 (IL-6) by ELISA kit (Thermo Fisher Scientific, Waltham, MA, USA) following the standard supplier protocol. The spectrophotometric readings were performed in a plate reader Infinite 200 Pro M Nano Plex (Tecan, Mannedorf, Svizzera).

### 2.8. Statistical Analysis

The results are presented as means of 3–7 replicates ± standard deviation. The statistical data analysis was performed by applying ANOVA with Tukey HSD post hoc test. Differences were considered statistically significant when *p* < 0.05. The data were processed using Microsoft Office Excel 2010 (Microsoft, Redmond, WA, USA) software.

## 3. Results

First, the antioxidant activity in essential oils was compared. The two samples of each category were analyzed: the essential oil without excipient, or pure essential oil (EO1), and essential oil with 1% of magnesium aluminometasilicate (EO2). The results are presented in Table 1. The range of essential oil concentration in this examination was from 0.2 to 20%. 

Both essential oils demonstrated similar antioxidant activity increasing in a concentration-dependent manner, except for some small fluctuations at 2% (EO2 had slightly higher antioxidant activity compared to EO1) and 20% (EO2 antioxidant activity was slightly lower than EO1). Both essential oil preparations at 10% concentration had higher than 50% antioxidant activity (more than half of DPPH radicals were bound).

Next in the study, the antioxidant activity of essential oil hydrolats was tested by using DPPH radical scavenging method. Hydrolat from EO1 was named EOH1, and from hydrolat from EO2 was named EOH2. The data are provided in Table 2.

At small quantities of up to 0.3 mL, the antioxidant activities of both hydrolat preparations were similar, but at 0.5 mL and 1 mL, the EOH1 antioxidant activity was significantly higher compared to that of EOH2. EOH1 at 1 mL had an antioxidant activity greater than 50%, and this activity level was similar to 5%–10% of EO1 activity. The results show that free radical scavenging activity of 0.2 mL of hydrolat is higher than that of 1% or less concentrated essential oil.

Summarizing the antioxidant activity results, magnesium aluminometasilicate did not improve, and even slightly decreased the antioxidant activity of nutmeg essential oil and its hydrolat.

Next in the study, antibacterial properties of nutmeg essential oil and hydrolats were investigated on 15 pathogenic clinical isolate strains by using a dilution range assay. The results are presented in Table 3.

EO1 only suppressed *Pasteurella multocida* growth, with the minimal concentration to achieve this effect being 0.2%. However, nutmeg essential oil with 1% of magnesium aluminometasilicate (EO2) had a broader effect. Next to *P. multocida*, it inhibited *E. faecalis* and *S. mutans*, and the efficient concentrations were rather low. A mere 0.5% was enough to completely suppress *E. faecalis*, and for the *S. mutans* strain even less than 0.1% was effective. In the case of EOH1 and EOH2, only the hydrolat with aluminometasilicate suppressed the growth of *S. mutans.* Thus, the results indicate that the excipient magnesium aluminometasilicate broadens the spectrum of antimicrobial activity of nutmeg essential oil.

One of the most important pharmacological activities of plant essential oils is related to anti-inflammatory properties. Nutmeg essential oil is also known for inflammation reducing activity [24]. Therefore, next in the study, we have assessed nutmeg seed essential oil and hydrolat preparations in a virus mimetic Poly I:C-induced inflammation in vitro model by using human fibroblast cell culture. Before starting the treatments, the general toxicity test of the oils and hydrolats was performed and LD_50_ doses as well as safe concentrations were established.

**Figure 1:**
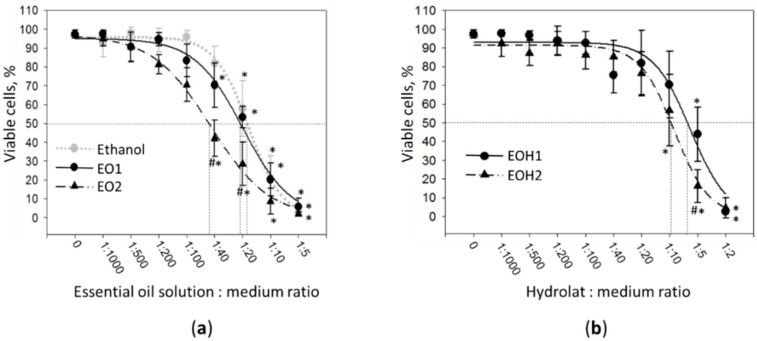
The effect of nutmeg essential oil ethanol solutions (**a**) and nutmeg essential oil hydrolats (**b**) on viability of cultured human fibroblasts. EO1—essential oil without excipient solution, EO2—essential oil with 1% magnesium aluminometasilicate solution, EOH1—hydrolat from EO1, and EOH2—hydrolat from EO2. In addition, 96% ethanol was assessed as solvent control for the essential oil. Punctured lines indicate the dilution ratios corresponding to LD_50_. *—statistically significant difference compared to untreated control, #—compared to EO1 in (**a**) or EOH1 in (**b**), respectively, when *p* < 0.05.

As indicated in Figure 1a, there were no significant difference in cell viability detected after treatment with both EO1 and EO2 essential oil solutions up to the dilution ratio 1:100. Further increases in concentration up to the dilution ratio 1:40 significantly decreased viability of the fibroblasts. After treatment with essential oil solutions at 1:40, the viability dropped from 97 ± 2% in control to 70 ± 12% in the case of EO1, and to 42 ± 10% in the case of EO2. At the dilution ratio of 1:5, the percentage of viable cells in the cultures was lower than 10% in the case of both essential oil preparations. The dilution ratios corresponding to LD_50_ calculated for EO1, EO2, and 96% ethanol were 0.047, 0.022, and 0.055, respectively. Thus, EO2 was significantly more toxic for the cells compared to EO1, and also to ethanol. In contrast, the toxicity pattern of EO1 was very close to that of ethanol, indicating there were no or very little toxic compounds in this essential oil preparation.

Evaluation of cell viability after 24 h treatment with nutmeg seed essential oil hydrolats revealed that both EOH1 and EOH2 were not toxic up to a dilution of 1:20 (Figure 1b). After cell incubation with 1:10 EOH2, the percentage of viable cells in the cultures decreased to 57 ± 19%, making a significant difference compared with the untreated control. A significant viability drop in EOH1 treatment series was achieved when the dilution ratio 1:5 was applied. The level of viable cells in this treated cultures was 44 ± 14%. After treatment with EOH1 and EOH2 at the ratio 1:2, nearly all cells in the cultures were found to be necrotic. The dilution ratios corresponding to LD_50_ calculated for EOH1 and EOH2 were 0.160 and 0.105, respectively. Toxicity evaluation of the hydrolats indicated that EOH2 is slightly more toxic compared to EOH1.

**Figure 2:**
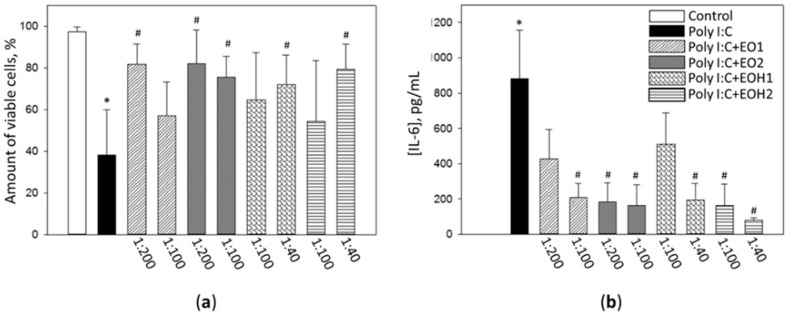
The effect of nutmeg seed essential oil ethanol solutions and the essential oil hydrolats on Poly I:C-treated human fibroblast cell viability (**a**) and cytokine IL-6 release from the cells (**b**). EO1—essential oil without the excipient solution, EO2—essential oil with 1% magnesium aluminometasilicate solution, EOH1—hydrolat from EO1, and EOH2—hydrolat from EO2. *—statistically significant difference compared to untreated control, #—compared to Poly I:C-only treatment, when *p* < 0.05; *n* = 5–7.

The next task in this work was to evaluate the efficiency of nutmeg seed essential oil and hydrolat preparations to reduce toxicity and signaling in viral inflammation in vitro model. To stimulate inflammatory response, human fibroblast cell culture was treated with 1 µg/mL virus double stranded RNR mimetic polyinosinic: polycytidylic acid (Poly I:C) for 24 h, with or without nutmeg seed essential oil solutions or hydrolats. After toxicity assessment, the dilution ratios selected for anti-inflammatory property testing were 1:200 and 1:100 for the essential oil solutions, and 1:100 and 1:40 for the hydrolats. Anti-inflammatory assessment results are presented in Figure 2.

After fibroblast cell culture treatment with 1 mg/mL Poly I:C, the amount of viable cells decreased by 59% (Figure 1a). Addition of nutmeg essential oil preparations to the cell culture medium increased cell viability in the Poly I:C-affected cultures. Statistically significant differences compared with Poly I:C samples were found after treatment with 1:200 EO1, 1:200 and 1:100 EO2, as well as 1:40 EOH1 and 1:40 EOH2. The percentages of viable nuclei in these samples were 81 ± 9%, 82 ± 16%, 76 ± 10%, 72 ± 14%, and 79 ± 12%, respectively. Thus, EO2 has demonstrated the highest cytoprotective capacity in a virus mimetic inflammation model.

Evaluation of the release of IL-6 to the incubation medium revealed that after 24 h of Poly I:C treatment, the level of this pro-inflammatory cytokine jumped from nearly a “zero” value to 883 ± 273 pg/mL (Figure 2b). Nutmeg essential oil preparations applied together with Poly I:C significantly reduced the concentration of IL-6 in the medium. The significant drop in the IL-6 level was in the samples incubated with 1:100 EO1, 1:200 and 1:100 EO2, 1:40 EOH1, and 1:100 and 1:40 EOH2. IL-6 concentration in these samples was found in the range between 162 ± 123 pg/mL (with 1:40 EOH2) and 206 ± 83 pg/mL (with 1:100 EO1). The assessment of IL-6 release indicates that both the solution of nutmeg seed essential oil with magnesium aluminometasilicate and the hydrolat from this essential oil are most efficient against Poly I:C-induced release of this inflammatory cytokine.

## 4. Discussion

The purpose of our study was to compare the biological activity of nutmeg seed essential oil and hydrolat without excipient and using magnesium aluminometasilicate as the excipient. To our knowledge, it is the first application of aluminometasilicate as an excipient in essential oil studies. Essential oils have strong antioxidant activity, and some of them are used as preservation agents protecting food or cosmetics from oxidation-induced spoilage [25,26]. Antioxidant activity studies help to elucidate essential oil capacity to protect food from free radical damage [27]. The DPPH radical scavenging method is widely used for this purpose because it is simple and cost-efficient, and gives reliable results. Therefore, it was selected to evaluate the essential oil and hydrolat preparations in our study. Our previous study [22] showed that magnesium aluminometasilicate had influence not only on the yield of essential oil, but also on its chemical composition. Magnesium aluminometasilicate significantly increased the quantity of sabinene, α-pinene, and limonene. Dai et al. (2013) study with *Wedella Prostrata* essential oil (containing 11.38% limonene and 10.74% α-pinene) had a lower antioxidant activity than 100 µg/mL limonene but a higher antioxidant activity than the pure α-pinene [28]. Such a result suggests that limonene is a more prominent antioxidant compared to α-pinene. Our nutmeg seed essential oil (EO2) had 11.66 ± 3.39% α-pinene and 4.91 ± 0.71% limonene [22]. Based on the results of the study where the presence of limonenen together with α-pinene resulted in higher antioxidant activity [28], we can predict that our EO2 has higher antioxidant activity than the pure α-pinene sample. The *Juniperus scopulorum* 10% essential oil had a 54.7% antioxidant activity (composition: sabinene 50.7%, α-pinene 3.23%, limonene 2.22%, cis sabinene hydrate 0.58%) [29]. Our study shows that 10% essential oils EO1 and EO2 have 61.01 ± 0.26% and 62.11 ± 0.43% antioxidant activity, respectively. Predominant compounds in EO2 were sabinene 61.42%, cis sabinene hydrate 0.3%, limonene 5.62%, and α-pinene 15.05%. The EO1 essential oil had more β-pinene [22], which could increase its antioxidant activity. Other authors have demonstrated nutmeg essential oil with higher antioxidant activity besides α-pinene had also β-pinene [27]. Misharina et al. (2009) have also studied antioxidant properties of nutmeg essential oil and found that 16.5% concentrated solution had approximately 50% antioxidant activity [30]. Such differences may be due to the distinct technique of the research and the variation in nutmeg seed material.

Hydrolats analyzed in our study had lower antioxidant activity compared to essential oils, most likely because of the lower concentrations of volatile compounds. We have searched the literature data, but could not find any studies about the antioxidant activity of nutmeg seed hydrolats so far. Hydrolats prepared from other plant sources had different antioxidant properties. For example, *Salvia officinalis* 0.1 g/mL had about 30% radical scavenging activity. The same concentration of *Rosmarinus officinalis* hydrolat had about 50% activity [31]. Our hydrolats (0.1 g/mL) had 31.43 ± 1.55% (EO1) and 27.24 ± 1.63% (EO2) antioxidant activity—the same as *Salvia officinalis*. Nutmeg seeds hydrolats at the highest concentration tested (0.5 g/mL) had 56.42% and 44.19% antioxidant activity (EOH1 and EOH2, respectively).

Essential oils are known for bioactive compounds with antibacterial activity, therefore they are used as antimicrobial agents in medicine, pharmacy, cosmetology, and other fields [18]. However, different essential oils affect microorganisms in distinct ways—some suppress gram-positive effects, others suppress gram-negative effects [19]. Also, the effective concentration of essential oils vary. There are various methods for determining antibacterial activity (the agar disk-diffusion method, antimicrobial gradient method, dilution methods, and other methods) [32]. Dilution methods are the simplest methods used to determine whether the essential oil suppresses the growth of bacteria or not [23]. There are many techniques and methods used for antimicrobial activity evaluation, and therefore, it is difficult to compare the results obtained from the different studies. In our study, the EO1 essential oil (0.2%) only suppressed *Pasteurella multocida*. The essential oil EO2 with a higher quantity of sabinene, α-pinene, and limonene [22] had antimicrobial activity against three pathogens. Next to *P. multocida*, it also prevented growth *E. faecalis* of and *S. mutans*. The increased efficiency of EO2 against pathogenic strains can be explained by the higher quantity of volatile compounds. Nurjanah et al.’s (2017) study showed (an in vitro disc diffusion antimicrobial activity method) that *Myristica fragrans* essential oil (60% concentration was used) from Central Java inhibited the largest areas [33]. The inhibition areas were from 12.96 mm to 16.79 mm, with the control at 0 mm (*S. aureus*, *S. dysenteriae*, *S. typhi*, and *S. epidermidis*). In the essential oil used for the above-mentioned study, sabinene, α-pinene, and β-pinene quantities were the highest out of all of the chemical compounds (the concentrations were 18.82%, 16.54%, and 13.82, respectively). The essential oil EO2 investigated in this study has a similar composition, meaning it could also be efficient against these pathogens at higher concentrations. In another study, the nutmeg essential oil with similar quantity of volatile compounds had a significant effect on the inhibition of the growth of *E. coli* and *S. aureus* [34]. In this study, we found that essential oil EA2 (0.5%) inhibited *E. faecalis*. This bacteria resides in infected canals of teeth and is often found in the oral cavity after tooth canal repair [35]. Repeated oral care products with chlorhexidine promotes the development of *E. faecalis* resistance [36]. Since EA2 showed activity against *E. faecalis*, nutmeg essential oil could be recommended as a safe protective component for oral care products in the future.

The investigation of human fibroblast cell culture affected by virus mimetic Poly I:C showed that nutmeg essential oils and hydrolats have an anti-inflammatory effect protecting cell viability and significantly reducing the release of cytokine IL-6. EO2 had a higher effect on preventing Poly I:C-induced necrosis and both EO2 and EOH2 more efficiently protected against IL-6 release compared to preparations without aluminometasilicate EO1 and EOH1. This is most likely due to the increased amount and content of active substances (sabinene, α-pinene, and limonene) in the preparations that is a result of the use of the excipient. α-Pinene significantly decreases the LPS-induced production of IL-6, TNF-α and nitric oxide in bacterial lipopolysaccharide (LPS)-treated macrophages [37]. Sabinene from *Oenanthe crocata* essential oil significantly inhibits nitric oxide production in LPS and IFNγ-treated macrophages [38]. Limonene has a significantly decreased manifestation of inflammatory signals in rat models of ulcerative colitis via regulation of iNOS, cyclooxygenase-2 (COX-2), PGE2, and ERK [39]. However, there are not many studies about the anti-inflammatory effect of nutmeg essential oil preparations. Zhang et al. have demonstrated the anti-inflammatory activity of nutmeg oil in complete Freund’s adjuvant-injected rats [16]. Their study shows that nutmeg oil is effective in inflammatory pain relief via inhibition of the COX-2 pathway and substance P release. Another in vivo study exploring carrageenan-induced paw edema in rats have also confirmed the anti-inflammatory properties of nutmeg oil [24]. To the best of our knowledge, there are no in vitro studies on virus-induced anti-inflammatory activity of nutmeg oil. Dewi et al. have found that *M. fragrans* seed ethanolic extract and pure quercetin extract from *M. fragrans* inhibited NO production and the release of inflammatory cytokines, such as TNF-α, IL-6, and IL-1β from bacterial LPS-stimulated murine macrophages (RAW 264.7) in a dose-dependent manner. The essential oil of *Monodora myristica* was found to inhibit inflammation-related lipoxygenase [40]. Because of the high content of bioactive volatile compounds, which have been widely studied and characterized by gas chromatographic techniques [41] the essential oils might be good candidates for inhalation treatment of respiratory tract infections. Most common respiratory infections are induced by respiratory viruses, such as influenza or respiratory syncytial virus [42]. As a result, we examined the anti-inflammatory efficiency of nutmeg essential oil preparations in a virus mimetic Poly I:C mediated inflammation. Fibroblasts are multifunctional cells that are responsible for support of other, more tissue-specific cell types, regeneration, wound healing, extracellular matrix production, and inflammatory response [43]. They significantly contribute to the response to infection by secreting cytokines for monocyte/macrophage attraction and their conversion to inflammatory phenotype [44]. The release of IL-6 is one of the key inflammatory signals causing activation of matrix metalloproteinases, macrophages, neutrophil production, and is also involved in autoimmune responses in the condition such as chronic arthritis, osteoporosis, and psoriasis [41,45,46,47,48].

The results of our study indicate that nutmeg essential oil preparations have anti-inflammatory properties that might be exploited further for treatment or prevention of viral inflammation-related pathologies, taking the recent emerging nanotechnological and nutraceutical approaches in the field into account [49,50,51]. However, to increase the applicability of these substances, more studies have to be performed analyzing the mechanism of action of the essential compounds contained in the preparations.

## 5. Conclusions

Nutmeg essential oil prepared with and without magnesium aluminometasilicate as an excipient has similar antioxidant activity. Nutmeg essential oil hydrolat prepared without excipient has a higher antioxidant activity compared to that with magnesium aluminometasilicate as an excipient.

Nutmeg essential oil with aluminometasilicate has extended antibacterial properties compared to the pure oil without additions. Both preparations prevent growth of *P. multocida* strain, but the oil with aluminometasilicate also inhibits *E. faecalis* and *S. mutans* (referent).

Nutmeg essential oil preparations with aluminometasilicate have stronger anti-inflammatory activity in Poly I:C-affected fibrolast cell culture. The oil with the excipient has a higher degree of cytoprotection from Poly I:C-induced necrosis, and both the oil and hydrolats with excipient more efficiently prevent IL-6 release compared to the preparations without aluminometasilicate.

The results show that the application of magnesium aluminometasilicate as an excipient in hydrodistillation could help to increase the biological activity of essential oil and hydrolats.

## Figures and Tables

**Table 1:** Antioxidant activity of nutmeg essential oils applied at different concentrations.

Sample	Essential Oil Concentration (%)						
	0.2	0.5	1	2	5	10	20
EO1	12.63 ± 0.53	16.34 ± 1.23	26.35 ± 0.88	30.58 ± 1.39	44.53 ± 0.84	61.01 ± 0.26	84.01 ± 0.78
EO2	12.65 ± 2.05	19.12 ± 2.24	27.03 ± 0.98	37.15 ± 0.80 *	44.92 ± 0.63	62.11 ± 0.43	72.71 ± 0.79 *

*—significant difference compared to EO1, *p* < 0.05, *n* = 3.

**Table 2:** Antioxidant activity of nutmeg essential oil hydrolats.

Sample	Hydrolat Quantity (mL)				
	0.1	0.2	0.3	0.5	1
EOH1	12.97 ± 1.25	31.43 ± 1.55	36.21 ± 3.20	48.09 ± 3.96	56.42 ± 3.23
EOH2	15.22 ± 5.14	27.24 ± 1.63	33.52 ± 2.11	36.55 ± 0.68 *	44.19 ± 1.09 *

*—significant difference compared to EOH1, *p* < 0.05, *n* = 3.

**Table 3:** Antimicrobial study results of nutmeg essential oil and its hydrolats.

Sample	Microorganisms														
	1	2	3	4	5	6	7	8	9	10	11	12	13	14	15
EO1	+	+	+	+	+	+	+	+	+	+	+	+	+	+	0.2
EO2	+	+	+	+	+	+	0.5	+	+	≤0.1	+	+	+	+	0.2
EOH1	+	+	+	+	+	+	+	+	+	+	+	+	+	+	+
EOH2	+	+	+	+	+	+	+	+	+	0.5	+	+	+	+	+

+ means the pathogens growth. 1. Klebsiella pneumoniae, 2. Salmonella enterica 24 SPn06, 3. Pseudomonas aeruginosa 17-331, 4. Acinetobacter baumanni 17-380, 5. Proteus mirabilis, 6. 6MRSA M87fox, 7. Enterococcus faecalis 86, 8. Enterococcus faecium 103, 9. Bacillus cereus 18 01, 10. Streptococcus mutans (referent), 11. Enterobacter cloacae, 12. Citrobacter freundii, 13. Staphylococcus epidermidis, 14. Staphylococcus haemolyticus, 15. Pasteurella multocida. Where the growth of bacteria were inhibited, minimal inhibitory concentrations were provided in %.
